# The Effect of Fasting on Human Metabolism and Psychological Health

**DOI:** 10.1155/2022/5653739

**Published:** 2022-01-05

**Authors:** Yiren Wang, Ruilin Wu

**Affiliations:** Department of Psychology, Beihang University, Beijing, China

## Abstract

Fasting is a prevalent approach to weight loss and is a feasible method for treating some diseases, such as type 2 diabetes. Meanwhile, the effects of intermittent fasting on health, aging, and disease process are hot issues and are of concern by researchers of multiple areas, even the public. This article introduces the effects of fasting on human lipid metabolism, glucose metabolism, protein metabolism, and neuroendocrine metabolism; demonstrates the metabolic conversion caused by fasting; and describes the effects of fasting on human psychological health, the relationship between mood regulation and glucose, and the emotional enhancing effect induced by fasting.

## 1. Introduction

Fasting means a restriction of the intake of solid foods. Based on traditional, cultural, or religious backgrounds, there are types of periodic fasting applied all over the world. In ancient medicine, fasting is a treatment method established since Hippocrates. Since then, it has been recommended by most older European medical schools for the treatment of acute and chronic diseases [1]. The food consumption frequency of modern people tends to have long daily energy intake periods and short fasting periods, and high-calorie diets and sedentary lifestyles affect the body's metabolism and increase the incidence of obesity, diabetes, cardiovascular disease, stroke, and dementia year by year [2]. Consequently, researchers have begun to explore the effects of different fasting strategies on the body to find dietary intervention programs that are suitable for controlling obesity. Fasting can be classified into short-term fasting, such as intermittent fasting (IF), and prolonged fasting (>8 days).

Intermittent fasting (IF) is a new type of dietary intervention. During the fasting period, IF requires the subjects not to consume any calorie-containing food, and “intermittent” highlights the characteristics of an alternating fasting time. IF can include alternate-day fasting, time-restricted feeding, whole-day fasting, and modified fasting methods. Alternate-day fasting (ADF) means that the fasting day and the feeding day alternate each day. The caloric intake on the fasting day is 25% of the daily caloric intake (approximately 2090 kJ), and the subjects can eat freely on the feeding day [3]. Time-restricted feeding means that subjects need to fast for the specified time within each day and then eat freely during the rest of the time. The times of fasting and eating within each day can be reasonably allocated according to personal preferences or living habits. Common time allocation methods are 16/8 (16 h fasting, 8 h free eating), 18/6 (18 h fasting, 6 h free eating), and 20/4 (20 h fast, 4 h free eating) [4]. The modified fasting method refers to prescribed fasting days each week; the intake of calories on the designated day is only approximately 1672~2508 kJ, and free eating occurs during the rest of the week, with plans such as 5 : 2 (2 days of limited calorie intake) and 4 : 3 (3 days of limited calorie intake) [4]. Ramadan fasting is a special type of time-restricted feeding; during Ramadan, people are not allowed to eat, drink, smoke or take medication during the daytime but are allowed to eat or drink from sunset until dawn [5, 6]. Compared with the Mediterranean diet or continuous caloric restriction (CR), IF does not restrict calorie intake during the ad libitum period, so it has better compliance and tolerance. This article demonstrates the effects of fasting on human metabolism and psychological health.

## 2. The Effect of Fasting on Human Metabolism

### 2.1. The Effect of Fasting on Human Lipid Metabolism

Studies have shown that alternate-day fasting over 8 to 12 weeks causes decreases in LDL cholesterol concentrations (20-25%) and triacylglycerol concentrations (15-30%), and increases in LDL particle size are often observed [7]. Similarly, alternate-day fasting trials of a 3- to 12-week duration appear to be effective at reducing total cholesterol (10%–21%) and triglycerides (14%–42%) in normal-weight, overweight, and obese humans. Whole-day fasting trials lasting 12 to 24 weeks also favorably improve blood lipids (5%–20% reduction in total cholesterol and 17%–50% reduction in triglycerides) [8]. The results of the above studies jointly suggest that IF can decrease blood lipids. A shift from preferential lipid synthesis and storage to the mobilization of fat typically occurs when glycogen in hepatocytes is depleted (12-36 hours after fasting starts), and the speed of lipolysis in adipose tissue increases and causes increased plasma levels of free fatty acids (FFAs) to produce increased fatty acid-derived ketones in the liver, kidney, astrocytes, and enterocytes as an energy supply [5, 9, 10]. In addition, all types of IF can promote thermogenesis caused by WAT browning by increasing the expression of thermogenesis genes. Brown adipose tissue plays a key role in energy homeostasis and thermogenesis. It mainly expresses uncoupling protein 1 (UCP1) to promote the uncoupling of the energy produced by mitochondrial oxidative phosphorylation and ATP synthesis, which ultimately leads to the energy produced being released in the form of heat, which increases energy expenditure and ultimately reverses the hyperlipidemia induced by a high-fat diet [11].

### 2.2. The Effect of Fasting on Human Glucose Metabolism

After an 8-week alternate-day fasting regimen, the fasting glucose of adults with obesity decreased significantly, and the insulin levels in the participants decreased although not significantly [12]. However, in another study, after an 8 h time-refrained feeding (ad libitum feeding between 10:00 and 18:00, water fasting between 18:00 and 10:00) for 12 weeks, the fasting glucose of adults with obesity showed no significant changes, and the insulin and HOMA-IR of the participants decreased, although not significantly [13].

In another study, after a 2-day severe intermittent energy restriction (IER) followed by 5 days of habitual eating for 12 weeks, the blood glucose of the participants who had type 2 diabetes or obesity was controlled well, and their HbA1c decreased [14]. Another study showed that after early time-restricted feeding (eTRF, 6 h feeding period, with dinner before 3 p.m.) for 5 weeks, the insulin sensitivity and *β* cell responsiveness of the participants with prediabetes were improved [15].

It can be seen from the studies above that the degree of influence of various IFs on glucose metabolism in obese individuals is inconsistent, depending on the fasting window period, the length of the period, and the baseline characteristics of the subjects.

### 2.3. The Effect of Fasting on Human Protein Metabolism

During fasting, protein in the body is oxidized and decomposed to produce energy. Amino acids are the most basic substances that constitute biological proteins, and they are related to life activities. They also have special physiological functions in the body and are one of the indispensable nutrients. Fasting can cause changes in the content and types of amino acids, and the length of the fasting time will affect the content and types of amino acids. Palou et al. studied the effects of plasma metabolic parameters in rats under fasting conditions for 24 hours. Plasma lactic acid, total amino acids, and the total amount of essential amino acids were significantly reduced starting from 3 hours of fasting. Glycerin, free fatty acids, *β*-hydroxybutyric acid, and acetoacetic acid increased significantly during fasting, while the contents of arginine, alanine, serine, threonine, aspartic acid, and aspartic acid and proline were significantly reduced. Several other amino acids were almost unchanged by fasting. The concentrations of lysine, leucine, isoleucine, and taurine underwent biphasic changes; their lowest content occurred at 6 hours after fasting, and there was a short recovery after 12 hours of fasting. The content of essential amino acids decreased significantly, more so than that of nonessential amino acids [16]. Another study showed that during the fasting period, in cases of a low concentration of urea, the ammonia concentration was significantly reduced, while the nitrogen concentration was relatively constant [17].

### 2.4. The Effect of Fasting on Human Neuroendocrine Metabolism

In a prospective study of obese subjects, fasting for more than 16 days resulted in substantial weight loss while reducing the baseline and exercise-induced serum norepinephrine, epinephrine, and dopamine concentrations [18]. In addition, prolonged fasting leads to an increase in the concentration of growth hormone glucagon and a decrease in the blood levels of thyrotropin and T3/T4 [19]. The release and turnover of serotonin will increase during a prolonged fasting period [20]. The plasma level of *β*-endorphin is significantly increased in subjects fasting for 5-10 days [21]. In rodents, fasting increases the expression of neuropeptide Y genes in specific brain regions [22]. In addition, catecholamines and glucocorticoids are released in large quantities during the first 7 days of fasting [1].

### 2.5. Intermittent Fasting and Metabolic Conversion

Glucose and fatty acids are the main energy sources for cells. After a meal, glucose is used as energy, and fat is stored in adipose tissue in the form of triglycerides. During fasting, triglycerides are broken down into fatty acids and glycerin to provide energy. Then, the liver converts fatty acids into ketone bodies, which provide the main energy source for many tissues, especially the brain [9]. In the fed state, the blood ketone body level is low, while in the fasting state, it increases within 8 to 12 hours in humans, reaching a level of 2 to 5 mM by 24 hours [23, 24].

## 3. The Effect of Fasting on Human Psychological Health

### 3.1. The Effect of Short-Term Fasting on Human Psychological Health

Some studies reported that short-term fasting can increase negative emotions (depression, anxiety, anger, irritability, fatigue, and tension) and decrease positive emotions and vitality [25–29]. In a two-day consecutive fasting study, lower positive mood, higher negative mood, and lower perceived work performance were observed, but the effects of fasting on positive mood and perceived work performance resulted from the consequent distraction, not the act of fasting or the result of hunger. This distraction might be a result of the attention required for fasting [25].

In contrast, some studies found that short-term fasting can cause mood enhancement, which is reflected by increased positive mood and vitality and decreased negative mood [28–33]. In an 18 h fasting among healthy women, they found that fasting can lead to increased irritability and increase positive affective experiences such as a sense of achievement, reward, pride, and control [28]. A 16 h fasting study showed that fasting may enhance fear extinction retention and prevent the return of fear, and this effect may persist for at least 6 months.

However, some studies have shown that there is no significant difference between fasting days and nonfasting days or between fasting subjects and nonfasting subjects in terms of positive or negative emotions [27, 29, 34, 35].

### 3.2. The Reason for the Different Outcomes among the Above Studies

There are some probable reasons for the different results among the above studies. First, strong religious beliefs can lead to positive effects on human physical and psychological health; for those who value their religious beliefs, fasting can be a pleasant and tolerant experience [31]. In contrast, fasting may bring negative emotions to those who do not have religious beliefs. Second, fasting is closely related to self-emotional control. On the one hand, fasting is a process that requires considerable cognitive effort, including self-emotional control, such as controlling the desire to eat and keeping a fast for a few days. On the other hand, successfully completing the fasting period may increase the feeling of self-control [28, 29]. Third, different assessment tools for psychological targets may lead to different outcomes. For instance, in the Ramadan fasting study in Germany, they used the visual analogue scale (VAS) and fatigue severity scale (FSS) to measure fatigue. The VAS results showed that fatigue first increased and then decreased, but the FSS results showed that fatigue continued to decrease during Ramadan fasting [30]. Fourth, previous fasting experience may cause different outcomes. A study showed that, compared to the group with previous fasting experience, the group with no previous fasting experience had more negative mood states, more stress, and less vitality [36]. Last, the initial baseline of the mental state is very important, and different initial baselines of the mental state may cause different outcomes. In a Ramadan fasting study, the group with a normal mood state at the initial baseline did not have significant differences between pre- and post-Ramadan in depression, anxiety, and stress. However, the group that showed depressive, anxious, and stressful effects at the initial baseline measurement had lower scores of depression, anxiety, and stress at the end of Ramadan fasting. Therefore, Ramadan may have a positive effect on people who are experiencing depression, anxiety, and stress [31].

### 3.3. The Effect of Prolonged Fasting on Human Psychological Health

In a 10-day complete fasting study, the trends of subjective sensations (depression, anxiety, and fatigue) were “U”- or “∩”-shaped curves, and the turning points of the curves were consistent with the inflection point of a change of the energy substrates from serum glucose to ketone, which suggests that the changes in psychological states are related to physiological factors [37].

## 4. The Connection between Blood Glucose and Emotional Self-Control

The mechanism behind the effects of fasting on human emotions remains unclear. Food restriction obviously affects energy intake, thereby affecting the availability of energy and, more specifically, the availability of glucose. Low blood and brain glucose levels are associated with a poor mood [25].

### 4.1. Glucose and Its Working Principle

Brain activity almost entirely relies on glucose as energy [38]. Glucose in the blood is metabolized in brain areas that need to perform specific tasks [39]. Glucose provides the energy for neurons to fire impulses to support cerebral function. Therefore, the brain needs adequate glucose to function effectively [40]. When blood glucose levels are severely low, brain function will be severely disturbed, resulting in a large number of cognitive and behavioral deficits, such as impaired coordination, blurred vision, amnesia, weird behaviors and personality changes, confusion, and anxiety. Relatively subtle changes in glucose can also have an important impact on thinking and behavior [40–43].

### 4.2. Glucose and Emotion Regulation

Emotions are a powerful response that sometimes may be highly destructive. Many people seek to control their emotions, especially in hiding or suppressing strong unpleasant emotional states. Emotion regulation relies on self-control energy, so suppressing or amplifying emotions will consume self-control resources, leading to a decline in self-control ability afterward [44]. Emotion regulation appears to be particularly sensitive to glucose [40]. It has been argued that coping with or regulating the emotional state of aversion (such as anxiety) may require relatively large amounts of glucose [45]. To a certain extent, people try to regulate their emotions and avoid negative emotions. Hypoglycemia can be seen as a disruption of emotion regulation, leading to an unreasonable continuous and inappropriate expression of emotional distress. Studies have shown that hypoglycemia is associated with high levels of anxiety, irritability, nervousness, and other negative emotions [45, 46]. Consistent with the view that emotion regulation is particularly sensitive to glucose, people with poor glucose tolerance show signs of poor emotional regulation. For instance, diabetic patients with particularly poor glucose tolerance are more likely to develop severe depression or anxiety disorders than diabetic patients with better glucose tolerance [47]. Diabetic patients are more likely to lose their temper and be emotionally unstable than nondiabetic patients [45, 48]. These findings are consistent with the overall pattern of low glucose or low glucose tolerance associated with poor mood regulation and the inability to eliminate unpleasant emotions.

Another possibility is that glucose may directly improve mood, and some published evidence may be consistent with this possibility. A study showed that compared to the placebo group, the group that drank a glucose solution had more positive emotions [49]. Some researchers assume that glucose may improve emotions by self-control when people are trying to regulate their emotions [40]. Therefore, a review of evidence on glucose and emotions shows that glucose may improve emotion regulation, but the exact physiological mechanisms of the effect of glucose levels on self-emotional control are currently unclear. Identifying these mechanisms may be a fruitful approach in future research.

## 5. Mechanisms of Fasting-Induced Emotional Enhancement

### 5.1. Physiological and Endocrine Mechanisms of Fasting-Induced Emotional Enhancement

Prolonged fasting is a strong physiological stimulus that can cause significant hormonal changes. It is not clear which factors trigger this neuroendocrine activation, but the decreased availability of brain glucose, the consumption of leptin and insulin, and the feeling of hunger may play an important role [50]. Besides, a study showed that Foxa2 might act as a metabolic sensor in neurons of the lateral hypothalamus, integrating metabolic signals, adaptive behaviors, and physiological responses [51].

Leptin levels may be a strong signal of biological adaptation of the organism to starvation and have been associated with mood disorders [52]. Studies have shown that fasting can lead to leptin depletion [53, 54], and leptin modulates brain reward circuitry by enhancing the value of behaviors incompatible with feeding [55]. A study showed that fasting-induced neuroendocrine activation is also associated with increased concentrations of norepinephrine, epinephrine, dopamine, and cortisol in urine and serum [56]. Similarly, in a prospective study of obese subjects, fasting for more than 16 days resulted in substantial weight loss while reducing the baseline and exercise-induced serum norepinephrine, epinephrine, and dopamine concentrations [18]. In addition, prolonged fasting is associated with an increase in the concentration of the growth hormone glucagon and a decrease in thyrotropin and blood T3/T4 levels [19].

### 5.2. Neurobiological Mechanisms of Fasting-Induced Emotional Enhancement

Studies have shown that changes in some neurotrophic factors and neurotransmitters caused by fasting may participate in fasting-induced emotional enhancement [20, 21, 57–59]. Fasting can stimulate neurogenesis and enhance synaptic plasticity, which can regulate pain sensations and enhance cognitive function and the antiaging ability of the brain. These beneficial effects may be associated with changes in neurotrophic factors and neurotransmitters [58].

Serotonin release and turnover can increase during prolonged fasting [20]. Increased output by the serotonergic system may cause an elevated mood and reduced pain sensitivity [1]. Studies on rats have shown that the availability of brain tryptophan and serotonin increased during fasting, which may explain the effects of fasting treatments in migraineurs [57]. In addition, brain-derived neurotrophic factors (BDNFs) caused by intermittent fasting may be related to central serotonergic regulation [59]. Furthermore, a research indicates that BDNF and serotonergic signaling have a reciprocal relationship, in which BDNF enhances the production and release of serotonin [60].

Another potential mechanism of mood enhancement is related to changes in the release of endogenous opioids caused by fasting. The plasma *β*-endorphin levels of subjects who fasted for 5 to 10 days increased significantly during the fasting period [21].

Some neuropeptides may also be related to mood enhancement. BDNF may be related to fasting-induced mood enhancement by enhancing the production and release of serotonin [20, 57, 59]. Other neuropeptides, such as orexin and neuropeptide Y, are also related to eating behavior, appetite, and mood regulation, and they are inhibited in response to feeding-related signals and released during fasting [1]. In rodents, fasting increases the expression of neuropeptide Y genes in specific brain regions. Neuropeptide Y acts through its spinal receptors to reduce spinal neuronal activity and the behavioral signs of inflammation and neuropathic pain [22]. Therefore, neuropeptide Y induced by fasting may have pain relief effects.

Overall, fasting may activate specific self-protective cellular stress resistance mechanisms to override any potentially harmful effects of increased glucocorticoids and catecholamines. In contrast, excessive feeding is related to chronic neuroendocrine activation, which promotes neuronal degeneration and impairs neurogenesis [1].

## 6. Summary and Prospect

In summary, fasting not only leads to negative emotional states such as irritability but also positive psychological experiences such as a sense of reward, accomplishment, pride, and control. However, the sample sizes of the studies above were generally too small. Before intermittent fasting is recommended to be widely implemented in the population for the purpose of improving human health, there are still many issues that need to be further studied and clarified, including the method of intermittent fasting (fasting time, duration, and frequency), applicable population (age, gender, weight, and health status), long-term compliance, and safety. Hence, it is necessary to conduct larger and longer-term studies to determine whether intermittent fasting or other modified fasting regimens can be a viable option for diet and lifestyle.
